# Antibiotic resistance of *Staphylococcus aureus* isolated from raw cow and goat milk produced in the Tiaret and Souk Ahras areas of Algeria

**DOI:** 10.14202/vetworld.2021.1929-1934

**Published:** 2021-07-27

**Authors:** Sofiane Tamendjari, Farida Afri Bouzebda, Lina Chaib, Hebib Aggad, Mohammed Ramdani, Zoubir Bouzebda

**Affiliations:** 1 Department of Veterinary Science, Institute of Agronomic and Veterinary Sciences, University of Souk Ahras, Algeria; 2 Laboratory of Animal Productions, Biotechnologies and Health, University of Souk Ahras, Algeria; 3 Laboratory of Hygiene and Animal Pathology, University of Tiaret, Algeria; 4 Department of Zoology and Animal Ecology, Scientific Institute, University Mohammed V of Rabat, Morocco

**Keywords:** antibiotic resistance, cow and goat raw milk, methicillin-resistant *Staphylococcus aureus*, *Staphylococcus aureus*

## Abstract

**Background and Aim:**

*Staphylococcus aureus* is a leading cause of infection in both humans and animals. Most livestock strains have shown antibiotic resistance to the many molecules used in veterinary therapeutics. This study aimed to assess the resistance patterns of these bacteria, we carried out our study in the Tiaret and Souk Ahras areas of Algeria.

**Materials and Methods:**

We collected 116 samples of bovine and goat milk to detect *S. aureus*. We used a selective media to isolate the strains, followed by biochemically identifying the isolates. We determined the susceptibility of the strains to antibiotic molecules using the disk diffusion method and confirmed the methicillin-resistant *S. aureus* (MRSA) with oxacillin minimum inhibitory concentration (MIC).

**Results:**

Our results showed that 26.72% of the samples were contaminated with *S. aureus*, and we recovered 31 isolates from the positive samples. We ascribed a high resistance profile to penicillin G (96.77%), fusidic acid (67.74%), and tobramycin (45.16%) and isolated 4MRSA strains.

**Conclusion:**

The presence of *S*. *aureus*, including MRSA strains in raw milk, can present a public health hazard, because these strains can cause widespread food poisoning. This finding will be useful to the veterinarians to choose an adequate treatment and to sensitize livestock breeders and milk producers to ensure the health of consumers.

## Introduction

*Staphylococcus aureus*, one of the most dangerous species in the genus, lives on the skin and mucous membranes of many animals and can cause diseases according to the infection source. *S. aureus* infections, which can result from direct contact with the environment or by consuming food products, can cause a variety of diseases, ranging in severity from slight skin infections to more severe diseases such as pneumonia, endocarditis, osteomyelitis, septicemia, or toxic shock syndrome. This broad range of clinical syndromes results from a variety of extracellular components, including surface proteins, capsules, enzymes, and toxins [1].

*S*. *aureus* produces a wide array of toxins, which are responsible for food poisoning in animals and can cause a variety of infections, including skin infections and mastitis in cows, goats, and sheep [2]. Mastitis in ruminants caused by clinical and sub-clinical forms can result in serious economic losses in the dairy sector by reducing production and lowering the quality of the milk [3].

The emergence of livestock-associated methicillin-resistant *S. aureus* (MRSA) is alarming because it is increasing worldwide and has a high risk of zoonotic transmission. Infection risk is especially high for professionals working in the agricultural industry [4] and probably for the community through the food chain [5]. The pathogenesis of *S. aureus* is aggravated by the acquisition of resistance to several antibiotics and other molecules with antimicrobial activity. In addition, MRSA is associated with patient care in hospitals and in the community. These strains are responsible for therapeutic failures and thus limit the choice of treatments for serious infections, causing increased costs for preventive and medical care [6].

This study aimed to assess the prevalence of *S. aureus* in raw cow and goat milk produced in the Tiaret and Souk Ahras regions of Algeria to evaluate the antibiotic resistance of these strains. This finding will be useful to the veterinarians to choose an adequate treatment and to sensitize livestock breeders and milk producers to ensure the health of consumers

## Materials and Methods

### Ethical approval

This study did not require any ethical approval from the University Animal Ethics Committee and was performed in accordance with Algerian laws and regulations on animal welfare.

### Study period and location

The study was conducted from March 2016 to November 2019. The study was conducted at the Laboratory of Hygiene and Animal Pathology, University of Tiaret and in Laboratory of Animal Productions, Biotechnologies and Health, University of Souk Ahras.

### Sample collection

We collected 116 samples, including 87 for bovine milk and 29 for goat milk. The bovine milk samples were collected from four farms in Tiaret (47 samples) and from three farms in Souk Ahras (40). However, the goat milk samples (29) were collected from only one goat farm in Ksar Chellala, Tiaret. All samples were collected aseptically in sterile boxes, transferred immediately to the laboratory with ice packs, and analyzed for the presence of *S. aureus*.

### *S. aureus* isolation and biochemical identification

Isolation of *S. aureus* was done by spreading 0.1 ml of dilution (10^−1^) on a Baird−Parker base (Conda Pronadisa, Spain), supplemented with egg yolk and potassium tellurite. Incubation of the plates was carried out at 37°C for 48 h [7].

From each positive sample, one colony with the typical aspects of *S. aureus* (black appearance, surrounded by a clear zone) was sub-cultured onto brain heart infusion agar (BHIB Conda, Pronadisa, Spain) to obtain a pure culture. The strains were submitted to Gram stain to confirm coccus morphology. The identification of isolates was completed using the following biochemical tests: Fermentation of mannitol, catalase, coagulase, and thermostable DNase.

### *S. aureus* isolates’ antimicrobial susceptibility

Antimicrobial susceptibility of strains was determined by the disk diffusion method on Mueller−Hinton agar (Conda, Pronadisa, Spain) according to the guidelines of the committee of the French Microbiology Society (FMS) [8] as well as the recommendations of the Algerian Antimicrobial Resistance Network [9]. The antibiotics disks used were from Liofilchem (Roeseto, Italy), and types and concentrations (μg) follow: Penicillin (PCN) G (10 UI), cefoxitin (FOX) (30), gentamicin (10), amikacin (30), tobramycin (TOB) (10), spiramycin (SP) (100), lincomycin (15), ofloxacin (5), tetracycline (TE) (30), trimethoprim/sulfamethoxazole (1.25/23.75), chloramphenicol (C) (30), fosfomycin (FOS) (50), fusidic acid (FA) (10), and novobiocin (NO) (30). The strains were classified as susceptible or resistant according to the FMS breakpoints [8].

### Beta-lactamase production

The detection of beta-lactamase (clover leaf test) was carried out for each strain with a PCN diameter of ≥29 mm [9].

### MRSA detection

MRSA was detected using oxacillin (OXA) agar screen, FOX and OXA disk diffusion tests, and determination of the minimal inhibitory concentrations (MICs).

### OXA-resistance agar screening

The bacteria suspension (adjusted to 0.5 McFarland turbidity standard) was inoculated on the OXA salt screen agar (Mueller–Hinton agar containing 4% NaCl and 6 μg/mL OXA). Plates were incubated at 37°C for 24 h, and any growth on the plate was regarded as methicillin resistance. Two *S. aureus* reference strains, ATCC 25923 and ATCC 43300, were used as negative and positive controls, respectively.

### MIC determination

MIC determination was carried out according to the guidelines of the U.S. Clinical and Laboratory Standards Institute (CLSI) [10].

### Statistical analysis

We performed the statistical analyses using Statistica 7 software (Statsoft, France).

## Results

### *S. aureus* prevalence

**Figure-1:**
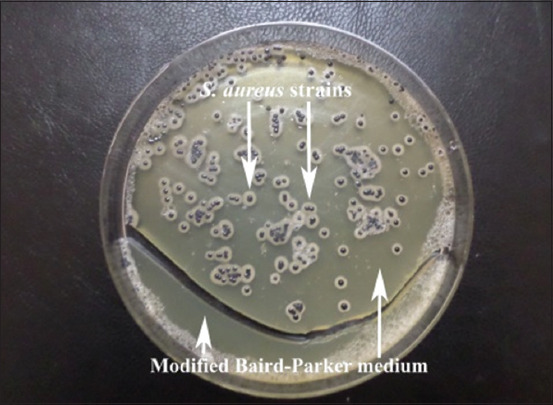
*Staphylococcus aureus* strains.

Of the 116 tested samples, 31 (26.72%) were contaminated with *S. aureus* (Figure-1), with 33.33% in cow milk (29/87 samples) and 6.89% in goat milk (2/29 samples). Cow milk showed a higher contamination rate as compared to goat milk (p<0.05), but we observed no significant difference between the cow milk of Tiaret and that of Souk Ahras (Figure-1).

### *S. aureus* isolates’ antimicrobial resistance

**Figure-2:**
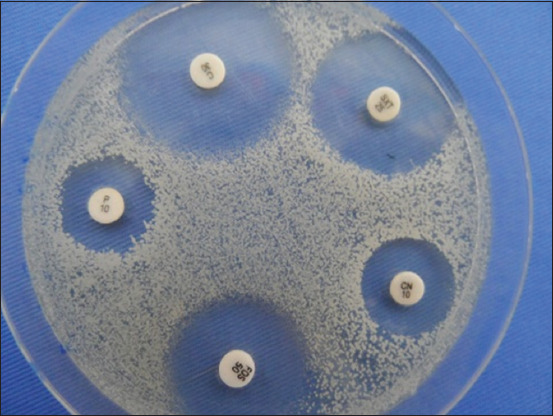
Result of antibiogram.

We found higher resistance rates for PCN G (96.77%), FA (67.74%), TOB (45.16%), TE (41.93%), and FOS (41.93%). We observed low resistance rates for C, NO, lincomycin, and SP (Table-1 and Figure-2).

**Table-1:** Frequencies of resistance to the antibiotics tested.

Families	Groups of antibiotics according to their significant differences	Resistance and intermediate to the antibiotic (%)	Sensitive to the antibiotic (%)
Beta-lactam	Penicillin^a^	96.77	3.23
	Cefoxitin^fg^	22.58	77.42
Aminoglycosides	Gentamicin ^de^	(41.93)	(58.07)
	Amikacin^d^	35.48	64.52
	Tobramycin^e^	45.16	54.84
Macrolides	Spiramycin^jk^	6.45	93.55
	Lincomycin^ij^	9.67	90.33
Quinolones	Ofloxacin^hi^	16.12	83.88
Tetracyclines	Tetracycline^de^	41.93	58.07
Sulfonamides	Trimethoprim/Sulfa methoxazol^gh^ ^gh^	(19.35)	(80.65)
Chloramphenicol	Chloramphenicol^ij^	6.45	93.55
Other molecules	Fosfomycin^e^	41.93	58.07
	Fusidic acid^b^	67.74	32.26
	Novobiocin^jk^	3.22	96.78

Significant difference, p=0.05

**Figure-3:**
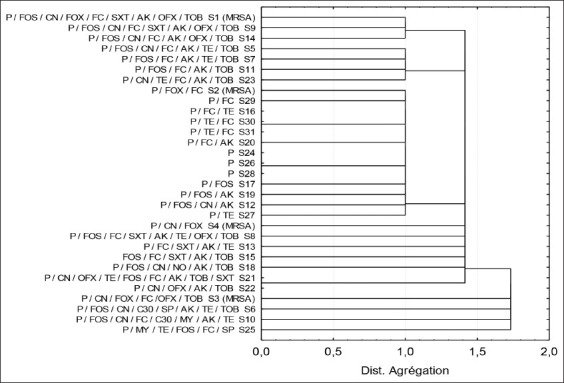
Classification of the identified strains of *S. aureu*s according to their antibiotic susceptibility.

Classification and relationship between the 31 identified *S. aureus* strains are indicated by a dendrogram showing the groups according to their degree of rapprochement (Figure-3).

### Beta-lactamase production

**Figure-4:**
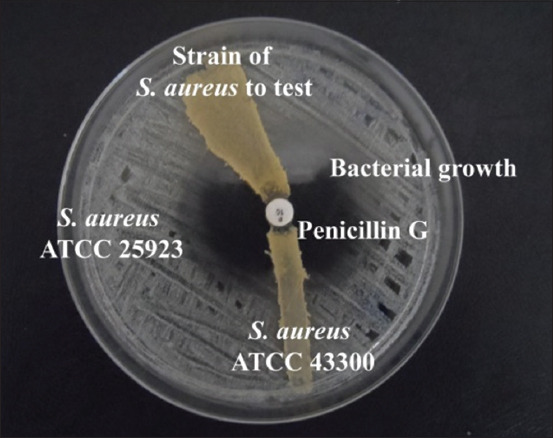
Beta-lactamase producing.

Almost all tested strains (90.32%) were producing the beta-lactamase enzyme (Figure-4).

### MRSA strain detection

**Figure-5:**
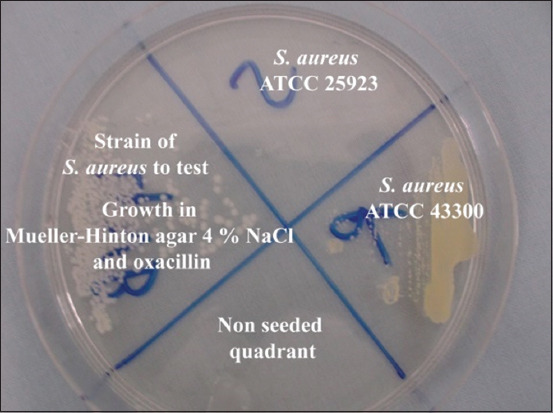
Methicillin-resistant *Staphylococcus aureus* test.

The FOX disk diffusion test revealed 4 MRSA strains, two isolated from cow milk and two from goat milk. These strains were confirmed by the OXA agar test and the MIC determination against OXA (Table-2 and Figure-5). All were producing beta-lactamases.

**Table-2:** MICs, phenotypes, and beta-lactamase producing of methicillin-resistant *Staphylococcus aureus* strains.

Strain	Origin	MIC (µg/mL)	Beta-lactamase producing	Antibiotic-phenotypes
S1	Raw cow’s milk	256	+	P/FOS/CN/FOX/FC/SXT/AK/OFX/TOB
S2	Raw cow’s milk	64	+	P/FOX/FC
S3	Raw goat’s milk	16	+	P/CN/FOX/FC/OFX/TOB
S4	Raw goat’s milk	16	+	P/CN/FOX

MIC=Minimum inhibitory concentration

## Discussion

Our results are in agreement with those reported by Chaalal *et al*. [11] in a recent study in Algeria, which has indicated an *S. aureus* contamination rate of 32.6% in raw milk. However, a high prevalence of *S. aureus* has been reported in Egypt [12] and India [13], with values of 75% and 68%, respectively.

We isolated two strains from 29 raw goat milk samples, with a rate of 6.89%. This result agrees with that of Ekici *et al*. [14] in Turkey, who isolated three strains from 25 goat milk samples, but is lower than that reported by Pexara *et al*. [15] in Greece (31.4%) and Bharathy *et al*. [13] in India (62.5%). Merz *et al*. [16] in Switzerland found no isolates of *S. aureus*. The prevalence of *S. aureus* can vary according to hygienic conditions and animal management [17]. Almost all isolated strains were beta-lactamase producing (96.77%). Our results differ from those announced by other studies indicating a low prevalence of these strains [18-20]. This high proportion of beta-lactamase-producing strains can be related to the overuse of PCN for the treatment of any infection at these farms, and the existence of these in the study area may be problematic for the treatment of *S. aureus* disease. The emergence of such strains in the environment requires monitoring [21]. We found that 12.9% of the isolated strains were resistant to methicillin, as were 3.44% of all the samples. Only a few studies have been conducted in Algeria to assess MRSA’s prevalence in milk and other foods. Chaalal *et al*. [11] have reported a rate of 21.5% in various types of foods, including raw milk, meat, and pastries. Other studies conducted in Algeria have found a lower prevalence, with values of 4.1%−13.6% in raw milk and traditional dairy products [22-24], but, to the best of our knowledge, no study has examined goat milk. The MRSA contamination rate in our study was higher than was that of many other studies [15,25,26] but was lower than that reported by Abd El Halem[27], with a value of 37.93%. This diversity in prevalence rates among studies can be related to attributes such as sample source, geographic origin, sensitivity of identification methods, sample quantity, inappropriate antimicrobial administration, preventive practices, production techniques, and sample storage and handling [24,28].

The MIC values for our results ranged from 64 µg/mL to 256 µg/mL, higher than those reported by other studies (Table-3) [29-33]. We found that 80.64% (Figure-3) of the isolated strains were resistant to at least three antibiotic families and are multi-resistant according to the CLSI [34] definition. No isolate was sensitive to all antibiotics. Our results do not agree with those of other studies in Algeria. Chaalal *et al*. [11] have reported that 33.3% of the *S. aureus* strains mainly isolated from raw milk and raw meat were multi-drug resistant, with 45.7% resistant to one antibiotic and 20.0% sensitive to all the antibiotics tested [11]. Matallah *et al*. [23] and Titouche *et al*. [22] have reported that 3.15% and 23.18% of their strains were multi-resistant. Similar findings have been obtained in Italy [25] and Russia [35].

**Table-3:** Minimum inhibitory concentration of certain strains of *Staphylococcus* aureus.

Study	Methicillin-resistant *Staphylococcus aureus* strain %	Origin	Minimum inhibitory concentration (µg/mL)
Our study	2.29	Raw cow’s milk	64-256
Our study	6.89	Raw goat’s milk	16
Moreno-Grúa *et al*. [29]	12.5	Commercial rabbits	8-256
Krupa *et al*. [30]	0.8	Chicken meat	64
Febler *et al*. [31]	37.2	Food and food products of poultry origin	4-32
Moon *et al*. [32]	2.8	Bovine mastitis	≤4-256
Lee [33]	6.41	Food animals	2-128

Food may be a vector for antibiotic-resistant bacteria and for the spread of antibiotic resistance, which can be transmitted through the consumption of food products of an animal origin [36]. Recent studies have highlighted the potential role of food in the spread of MRSA lineages in humans [37], and *S. aureus* is considered an important cause of zoonotic diseases and a potential source of transmission of the MRSA strains between livestock and humans through handling and consuming contaminated food [38].

## Conclusion

In this study, we report for the 1^st^ time the presence of MRSA in raw goat milk in Algeria. However, bovine milk was more contaminated by *S. aureus* than was goat milk. The high presence of multidrug-resistant *S. aureus*, including the MRSA strains, raises questions about the persistent use of antibiotics as the *a priori* treatment for udder and other infections on the farm. Our results indicate the need for continuous monitoring and improvement of the hygienic quality of raw milk by ensuring proper handling and production to reduce the spread of multidrug-resistant bacteria to foods of animal origin.

## Authors’ Contributions

ST and LC: Sample collection, microbiological analysis of samples and supply of certain products. ST: Writing of the manuscript and statistical analysis. FAB: Follow-up of the study, supply of some products, and correction of the manuscript. HA: Follow-up of the study and supply of some products. MR and ZB: Follow-up of the study and correction of the manuscript. All authors have read and approved the final manuscript.
